# Factors associated with the quality of conflict management among anesthesia technicians

**DOI:** 10.1192/j.eurpsy.2024.1240

**Published:** 2024-08-27

**Authors:** A. Ghenim, M. Kahloul, I. Kacem, A. Aloui, A. Chouchane, M. Ajmi, W. Naija, M. Maoua, N. Mrizak

**Affiliations:** ^1^Occupational Medicine Department, Farhat Hached Academic hospital; ^2^Sahloul Academic Hospital, Anesthesia and Intensive Care Department, Sousse, Tunisia

## Abstract

**Introduction:**

A conflict arises when one or more individuals, groups or organizations disagree, creating internal or external tensions that can cause damage. This is particularly serious in operating theatres, where decisions involving life or death are common. Indeed, in this particular context, the multiplicity of stakeholders, the divergence of opinions and decisions related to patient care, the frequency of critical situations, stress and the limitation of resources are established causes of disagreement and tension.

**Objectives:**

To identify factors associated with the alteration of conflict management quality among anesthesia and resuscitation technicians (ART).

**Methods:**

This is an observational, multicenter, cross-sectional and analytical study, enrolling all ART exercising at the two teaching hospitals of Sousse (Tunisia) over a two month period(March 1, 2022 to April 30, 2022). Conflict management was assessed using the Conflict Handling Style Scale.

**Results:**

Our study involved 50 participants, only eight of whom reported having had previous training in communication and conflict management. Conflicts in the hospital were rated frequent to very frequent by 58% of participants. Task conflicts were the most reported (74%). The main causes of conflict were lack of leadership (60%), unequal distribution of tasks (42%) and workload (28%). The main repercussions of the conflicts were the delay in patients care (60%), therapeutic errors (42%), and the cancellation or postponement of some acts (34%). The main factors associated with impaired conflict management abilities were age<40 years (p=0,03), tobacco consumption (p=0,001), and number of dependent children<2 p<10-^3^).

**Conclusions:**

In light of our results, it would be useful and urgent to develop the soft skills of our human resources, particularly in terms of communication and conflict management.

**Disclosure of Interest:**

None Declared

